# Cytotoxic Compounds from *Belamcanda chinensis* (L.) DC Induced Apoptosis in Triple-Negative Breast Cancer Cells

**DOI:** 10.3390/molecules28124715

**Published:** 2023-06-12

**Authors:** Ya-Ping Guo, Peng Yi, Qi-Qi Shi, Rui-Rui Yu, Jin-Hui Wang, Chen-Yang Li, Hai-Qiang Wu

**Affiliations:** 1 Guangdong Key Laboratory for Biomedical Measurements and Ultrasound Imaging, National-Regional Key Technology Engineering Laboratory for Medical Ultrasound, School of Biomedical Engineering, Shenzhen University Medical School, Shenzhen 518060, China; guoyaping_0816@163.com; 2 School of Pharmaceutical Sciences, Shenzhen University Medical School, Shenzhen 518060, China; 15115433780@163.com (P.Y.); daqiqi_818@163.com (Q.-Q.S.); 15220201716@163.com (R.-R.Y.); 3 School of Pharmacy, Harbin Medical University, Harbin 150081, China; wangjinhui@hrbmu.edu.cn

**Keywords:** *Belamcanda chinensis*, iridal-type triterpenoids, triple-negative breast cancer, apoptosis

## Abstract

Four compounds (**1**, **5**, **7**, and **8**) were first isolated from the genus *Belamcanda Adans*. nom. conserv., and six known compounds (**2**–**4**, **6**, **9**, and **10**) were isolated from the *rhizome* of *Belamcanda chinensis* (L.) DC. Their structures were confirmed by spectroscopic data. Herein, compounds **1**–**10** were rhapontigenin, trans-resveratrol, 5,7,4′-trihydroxy-6,3′,5′-trimethoxy-isoflavone, irisflorentin, 6-hydroxybiochannin A, iridin S, pinoresinol, 31-norsysloartanol, isoiridogermanal, and iristectorene B, respectively. All compounds were evaluated for their antiproliferative effects against five tumor cell lines (BT549, 4T1, MCF7, MDA-MB-231, and MDA-MB-468). Among them, compound **9** (an iridal-type triterpenoid) showed the highest activity against 4T1 and MDA-MB-468 cells. Further studies displayed that compound **9** inhibited cell metastasis, induced cells cycle arrest in the G1 phase, exhibited significant mitochondrial damage in 4T1 and MDA-MB-468 cells including excess reactive oxygen species, decreased mitochondrial membrane potential, and induced 4T1 and MDA-MB-468 cell apoptosis for the first time. In summary, these findings demonstrate that compound **9** exerts promising potential for triple-negative breast cancer treatment and deserves further evaluation.

## 1. Introduction

*Belamcanda chinensis* (L.) DC is the sole species in the genus *Belamcanda Adans*. nom. conserv., native to Southeast Asia (Bhutan, China, Japan, Korea, Myanmar, the Philippines, Thailand, the Ussuri region of Russia, and Vietnam), and it is widely distributed in Hubei, Henan, Jiangsu, and Anhui in China [1]. The *rhizome* of *Belamcanda chinensis* (L.) DC was introduced as a medicine in the first Chinese monograph on herbal medicines, *Shennong’s Classic of Materia Medica* [2]. It is known as “She Gan” in China, and its characteristics are that it is bitter and cold, related to the lungs, with sinking and dispersing action, and descending lung “qi”. It treats a sore throat by eliminating heat, toxins and phlegm, dyspnea, dissolves sputum, and relieves wheezing caused by the accumulation of heat and phlegm in the lungs [3,4,5,6,7].

To date, *Belamcandae chinensis rhizoma* is rich in a variety of flavonoids, stilbenoids, and triterpenoids. Flavonoids mainly contain flavones (hispidulin, isorhamnetin, rhamnazin, and so on) [7,8,9] and isoflavonoids (tectorigenin, irigenin, iristectorigenin A, and so on) [9,10,11,12]. Stilbenoids mainly consist of resveratrol, shegansu B, and so on [12,13]. In addition, triterpenoids, in particular iridal-type triterpenoids, are also characteristic constituents whose representative compounds include iridobelamal A [14,15], epianhydrobelachinal, belamcanoxide B [16], seconoriridone A [17,18], 3-*O*-decanoyl-16-*O*-acetyl-isoiridogermanal [19], belamchinenin A [17], and belamcandaoids A−N [20]. Moreover, quinones (belamcandones A−D) [1], and simple phenols (belallosides A and B and so on) [21] have been reported in *Belamcandae chinensis rhizoma.*

In recent decades, the pharmacological activities of *Belamcandae chinensis rhizoma* and isolated compounds have shown anti-tumor [22,23,24], anti-inflammatory [25,26,27,28,29], antioxidative [30,31,32], antimutagenic [33,34,35,36], phytoestrogenic [37,38], antidiabetic [10,39,40,41,42], antifungal [43], and antiviral [24] effects and so on. Furthermore, anti-tumor activities focusing on cervical cancer [44], human osteosarcoma cells [45], non-small-cell lung cancer [46], gastric cancer, colon cancer [47], prostate cancer [48], malignant testicular germ cell carcinoma [49], and the main anti-tumor compounds from *Belamcandae chinensis rhizoma* belong to isoflavonoids [23]. Nonetheless, triple-negative breast cancer cells (TNBC) are not known to be susceptible to triterpenoids, especially iridal-type triterpenoid-mediated cytotoxicity. A detailed investigation of specific constituents of *Belamcandae chinensis rhizoma* and their mode of action has not been provided with triple-negative breast cancer cells in any literature. Consequently, we aim to elucidate the mechanism of anti-triple-negative breast cancer of iridal-type triterpenoids isolated from *Belamcandae chinensis rhizoma*, so as to provide a theoretical basis for the further development and utilization of *Belamcandae chinensis rhizoma*.

In this study, we discovered four compounds (**1**, **5**, **7**, and **8**) first isolated from the genus *Belamcanda Adans*. nom. conserv. and six known compounds (**2**–**4**, **6**, **9**, and **10**), and their structures were established by spectroscopic analyses. Herein, compounds **1**–**10** are rhapontigenin, trans-resveratrol, 5,7,4′-trihydroxy-6,3′,5′-trimethoxy- isoflavone, irisflorentin, 6-hydroxybiochannin A, iridin S, pinoresinol, 31-norsysloartanol, isoiridogermanal, and iristectorene B, respectively. Furthermore, all isolates were tested for antiproliferative effects against five kinds of tumor cell lines (BT549, 4T1, MCF7, MDA-MB-231, and MDA-MB-468). Several compounds, including compound **9**, exhibited significant cytotoxicity in five cell lines, especially for the 4T1 and MDA-MB-468 cell lines. Subsequently, compound **9** (iridal-type triterpenoids) was examined in this study for the first time for its effect on proliferation, metastasis, cell cycle, apoptosis, mitochondrial membrane potential (MMP) collapse, and reactive oxygen species (ROS) formation in 4T1 and MDA-MB-468 cell lines.

## 2. Results and Discussion

### 2.1. Identification of Compounds ***1***–***10***

**Figure 1:**
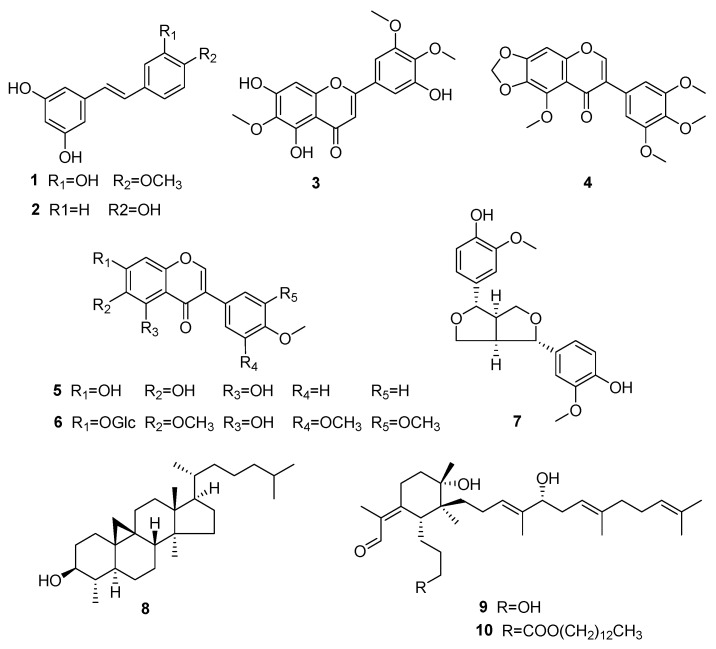
The structure of compounds **1**–**10**.

*Rhapontigenin* (**1**): colorless crystallization, ^1^H NMR (500 MHz, MeOD) *δ*_H_: 7.11 (1H, d, *J* = 1.9 Hz, H-2), 6.99–6.96 (1H, m, H-b), 6.95 (d, *J* = 2.0 Hz, 1H, H-2), 6.83 (1H, d, *J* = 16.3 Hz, H-a), 6.78 (1H, d, *J* = 8.1 Hz, H-5), 6.48 (2H, d, *J* = 2.1 Hz, H-2′ and H-6′), 6.18 (1H, t, *J* = 2.2 Hz, H-4′), 3.91 (3H, s, −OCH_3_).^13^C NMR (126 MHz, MeOD) *δ*c: 159.6 (C-3′, C-5′), 149.2(C-3), 147.6 (C-4), 141.3 (C-1′), 131.0 (C-1), 129.7 (C-*α*), 127.3 (C-*β*), 121.2 (C-6), 116.3 (C-5), 110.4 (C-2), 105.8 (C-2′ and C-6′), 102.7 (C-4′), 56.4 (OMe). All presented data of **1** were identical to rhapontigenin [50]. Hence, compound **1** was elucidated as rhapontigenin (Figure 1).

*Trans-Resveratrol* (**2**): colorless powder, ^1^H-NMR (500 MHz, MeOD) *δ*_H_: 7.37–7.34 (2H, m), 6.96 (1H, d, *J* = 16.3 Hz, H-1), 6.81 (1H, d, *J* = 16.4 Hz, H-2), 6.77 (2H, d, *J* = 8.6 Hz, H-3′, H-5′), 6.46 (2H, d, *J* = 2.2 Hz, H-2″, 6″), 6.17 (1H, t, *J* = 2.2 Hz, H-4″). ^13^C-NMR (126 MHz, MeOD) *δ*c: 159.6 (C-3″, C-5″), 158.4 (C-4′), 141.3 (C-1″), 130.4 (C-1′), 129.4 (C-1), 128.8 (C-2′, C-6′), 127.0 (C-2), 116.5 (C-3′, C-5′), 105.8 (C-2″, C-6″), 102.6 (C-4″). All NMR data of **2** were consistent with trans-resveratrol [51]. Consequently, compound **2** was established as trans-resveratrol (Figure 1).

*5,7,4′-trihydroxy-6,3′,5′-trimethoxyisoflavone* (**3**): yellowish crystallization, ^1^H-NMR (600 MHz, DMSO-d6) *δ*_H_: 13.08 (1H, s, 5-OH), 10.79 (1H, s, 7-OH), 8.54 (1H, s, 4′-OH), 8.41 (1H, s, H-3), 6.85 (2H, s, H-2′, H-6′), 6.51 (1H, s, H-8), 3.79 (6H, s, 3′-OMe, 5′-OCH_3_), 3.75 (3H, s, 6-OCH_3_). ^13^C-NMR (151 MHz, DMSO-d6) *δ*c:180.5 (C-4), 157.5 (C-7), 154.6 (C-2), 153.3 (C-5), 152.7 (C-9), 147.7 (C-3′, 5′), 135.8 (C-4′), 131.4 (C-6), 121.9 (C-3), 120.7 (C-1′), 106.8 (C-2′), 104.9 (C-10), 93.9 (C-8), 60.0 (6-OCH_3_), 56.1 (3′, 5′-OCH_3_). The NMR signals of **3** were the same as 5,7,4′-trihydroxy-6,3′,5′-trimethoxyisoflavone [52]. Accordingly, the structure of **3** was identified as 5,7,4′-trihydroxy-6,3′,5′-trimethoxyisoflavone (Figure 1).

*Irisflorentin* (**4**): colorless powder, ^1^H-NMR (500 MHz, MeOD) *δ*_H_: 8.14 (1H, s, H-2), 6.84 (2H, s, H-2′, H-6′), 6.81 (1H, s, H-8), 6.13 (2H, s, -O-CH_2_-O-), 4.08 (3H, s, 5-OMe), 3.88 (3H, s, 3′,5′-OMe), 3.81 (3H, s, 4′-OMe). ^13^C-NMR (126 MHz, DMSO-d6) *δ*c: 173.7 (C-4), 153.9 (C-5), 152.7 (C-9), 152.5 (C-3′, C-5′), 152.1 (C-2), 140.5 (C-7), 137.2 (C-4′), 136.0 (C-6), 127.5 (C-1′), 124.1 (C-3), 113.2 (C-10), 106.8 (C-2′), 102.7 (-O-CH_2_-O-), 93.7 (C-8), 60.8 (5-OCH_3_), 60.1 (4′-OCH_3_), 56.0 (3′, 5′-OCH_3_). All NMR signals of **4** were similar to irisflorentin [53]. Thus, the structure of **4** was proposed as irisflorentin (Figure 1).

*6-hydroxybiochannin A* (**5**): colorless needle crystallization, ^1^H-NMR (500 MHz, MeOD) *δ*_H_: 8.03 (1H, s, H-2), 7.36 (2H, d, *J* = 7.8 Hz, H-2′, 6′), 6.84 (2H, d, *J* = 7.9Hz, H-3′, 5′), 6.42 (1H, s, H-8), 3.86 (3H, s, -OCH_3_).^13^C-NMR (151 MHz, MeOD) *δ*c: 182.5 (C-4), 158.9 (C-7), 158.8 (C-5), 155.0 (C-4′), 154.9 (C-2), 154.5 (C-9), 132.8 (C-6), 131.4 (C-2′), 124.2 (C-1′), 123.3 (C-3), 116.2 (C-3′), 106.6 (C-10), 95.0 (C-8), 60.9 (-OCH_3_). All presented data of **5** were in accordance with 6-hydroxybiochannin A [54]. Therefore, the structure of **5** was determined as 6-hydroxybiochannin A (Figure 1).

*Iridin S* (**6**): colorless powder, ^1^H-NMR (600 MHz, DMSO-d6) *δ*_H_: 12.88 (1H, s, 5-OH), 8.56 (1H, s, H-2), 6.92 (1H, s, H-8),6.90 (1H, s, H-2′, H-6′), 5.46 (1H, d, *J* = 5.2 Hz, H-2″), 5.16 (1H, d, *J* = 4.7 Hz, H-1″), 5.11–4.63 (5H, H-2″~H-6″), 3.81 (3H, s, 3′-OMe, 5′-Ome), 3.77 (3H, s, 6-Ome), 3.70 (3H, s, 4′-Ome).^13^C-NMR (151 MHz, DMSO-d6) *δ*c: 180.5 (C-4), 156.7 (C-7), 155.7 (C-2), 152.9(C-5), 152.7(C-9), 152.4 (C-3′,C-5′), 137.6 (C-4′), 132.6 (C-6), 126.2 (C-3), 122.0 (C-1′), 106.7 (C-2′, C-6′), 106.5 (C-10), 100.1 (C-1″), 94.1 (C-8), 77.3 (C-3″), 76.7 (C-5″), 73.1 (C-2″), 69.7 (C-4″), 60.7 (3′, 5′-OCH_3_), 60.3 (4′-OCH_3_), 60.1 (C-6″, 5-OCH_3_)), 56.0 (6-OCH_3_). The NMR signals of **6** were in accordance with iridin S [55]. Therefore, the structure of **6** was assigned as iridin S (Figure 1).

*Pinoresinol* (**7**): colorless powder, ^1^H-NMR (500 MHz, DMSO-d6) *δ*_H_: 6.89 (2H, s, H-2′, H-2″), 6.74 (4H, q, *J* = 8.0 Hz, H-5′, H-5″, H-6′, H-6″), 4.60 (2H, s, H-2, H-6), 4.15–4.07 (2H, m, H-4eq, H-8eq), 3.76 (6H, s, 2 × -OCH_3_), 3.72 (2H, d, *J* = 9.0 Hz, H-4ax, H-8ax), 3.03 (2H, s, H-1, H-5). ^13^C-NMR (126 MHz, DMSO-d6) *δ*c: 147.5 (C-3′, C-3″), 145.9 (C-4′, C-4″), 132.2 (C-1′, C-1″), 118.6 (C-6′, C-6″), 115.1 (C-5′, C-5″), 110.4 (C-2′, C-2″), 85.2 (C-2, C-6), 70.9 (C-4, C-8), 55.6 (2 × -OCH_3_), 53.6 (C-1, C-5). The NMR signals of **7** were the same as pinoresinol [56]. As a consequence, the structure of **7** was established as pinoresinol (Figure 1).

*31-norsysloartanol* (**8**): colorless flaky crystallization, ${\left[\alpha \right]}_{D}^{25}\;$= +43 (c 0.05, MeOH). ^1^H-NMR (600 MHz, CDCl_3_) *δ*_H_: 3.21 (1H, m, *J* = 10.9, 9.3, 4.7 Hz, *α*-H), 2.01–1.93 (m, 2H), 1.70 –1.48 (m, 10H), 1.42–1.24 (m, 10H), 1.20–1.08 (m, 6H), 0.98–0.96 (6H, m, 2 × -CH_3_), 0.89–0.85 (m, 12H), 0.58 (qd, *J* = 12.8, 2.9 Hz, 1H), 0.38 (1H, d, *J* = 3.9 Hz, H-19), 0.14 (1H, d, *J* = 4.1 Hz, H-19).^13^C-NMR (151 MHz, CDCl_3_) *δ*c: 76.7 (C-3), 52.5 (C-17), 49.0 (C-14), 47.0 (C-8), 45.4 (C-13), 44.7 (C-4), 43.5 (C-5), 39.7 (C-24), 36.6 (C-22), 36.3 (C-20), 35.5 (C-12), 35.0 (C-2), 33.0 (C-15), 30.9 (C-1), 29.7 (C-10), 28.3 (C-25), 28.2 (C-7), 27.4 (C-19), 27.1 (C-16), 25.3 (C-11), 24.8 (C-6), 24.3 (C-23), 23.7 (C-9), 23.0 (C-27), 22.7 (C-25), 19.3 (C-28), 18.5 (C-21), 17.9 (C-18), 14.5 (C-29). The NMR signals of **8** were identical to 31-norsysloartanol [57]. Hence, compound **8** was elucidated as 31-norsysloartanol (Figure 1).

*Isoiridogermanal* (**9**): yellow oil, ${\left[\alpha \right]}_{D}^{25}\;$= +46 (c 0.05, MeOH). ^1^H-NMR (600 MHz, CDCl_3_) *δ*_H_: 10.21 (1H, s, H-1), 5.20 (1H, t, *J* = 7.0 Hz, H-14), 5.09 (1H, t, *J* = 7.1 Hz, H-18), 5.00 (1H, t, *J* = 7.2 Hz, H-22), 3.86 (1H, t, *J* = 7.0 Hz, H-16), 3.51 (2H, td, *J* = 6.6, 2.3 Hz, H-3), 3.37 (1H, d, *J* = 17.7 Hz, H-6), 2.72–2.62 (1H, m, H-5), 2.56 (1H, ddt, *J* = 13.7, 4.2, 2.0 Hz, H-9), 2.20 (2H, td, *J* = 7.1, 4.3 Hz, H-15, H-23), 2.08–2.01 (2H, m, H-11), 1.95 (4H, dd, *J* = 9.6, 5.3 Hz), 1.82 (3H, d, *J* = 1.1 Hz), 1.68 (3H, s), 1.61 (3H, s), 1.58 (3H, s, H-29), 1.52 (3H, s, H-30), 1.36–1.17 (3H, m), 1.12 (6H, d, *J* = 4.3 Hz, H-27, H-26).^13^C-NMR (151 MHz, MeOD) *δ*c: 192.1 (C-1), 166.5 (C-7), 137.8 (C-15), 137.4 (C-19), 134.0 (C-2), 132.1 (C-23), 127.4 (C-14), 125.4 (C-22), 121.7 (C-18), 78.7 (C-16), 75.2 (C-10), 63.2 (C-3), 46.1 (C-11), 45.0 (C-6), 40.9 (C-20), 38.2 (C-4), 37.9 (C-9), 34.5 (C-17), 33.7 (C-12), 28.0 (C-21), 27.8 (C-5), 26.0 (C-27), 25.9 (C-24), 25.0 (C-8), 22.8 (C-13), 18.4 (C-26), 17.8 (C-30), 16.4 (C-29), 11.3 (C-28), 10.9 (C-25). The NMR data of **9** were consistent with isoiridogermanal [16,58], Consequently, compound **9** was established as isoiridogermanal (Figure 1).

*Iristectorene B* (**10**): yellow oil, ${\left[\alpha \right]}_{D}^{25}$= −22 (c 0.05, MeOH).^1^H-NMR (600 MHz, CDCl_3_) *δ*_H_: 10.14 (1H, s, H-9), 5.32 (1H, d, *J* = 15.9 Hz, H-3′, 5.22 (1H, s, H-7′), 5.04 (2H, s, H-7′, H-11′), 3.99 (1H, s, H-3″), 3.90 (1H, s, H-5′), 3.27 (1H, d, *J* = 10.6 Hz, H-3), 2.52 (1H, s, H-5), 2.25 (2H, s, H-6, H-5′), 2.04 (2H, s, H-9′, H-10′).^13^C-NMR (126 MHz, CDCl_3_) *δ*c: 190.1 (CHO), 174.2 (C=O), 162.9 (C-4), 138.8 (C-8′), 137.2 (C-4′), 133.5 (C-8), 131.8 (C-12′), 125.5 (C-3′), 124.3 (C-11′), 120.1 (C-7′), 76.9 (C-5′), 75.1 (C-1), 64.4 (C-3″), 44.9 (C-2), 43.5 (C-3), 40.0 (C-9′), 37.2 (C-6), 37.0 (C-1′), 34.6 (C-6′), 34.5 (C-5″), 32.1 (C-15″), 29.8 (C-11″), 29.8 (C-12″), 29.8 (C-13″), 29.6 (C-10″), 29.5 (C-9″), 29.5 (C-14″), 29.3 (C-8″), 28.8 (C-2″), 26.7 (C-10′), 26.4 (C-7), 25.9 (C-7), 25.2 (C-13′), 25.1 (C-6″), 24.0 (C-5), 22.8 (C-16″), 21.9 (C-2′), 18.1 (C-11), 17.9 (C-16′), 16.4 (C-15′), 14.3 (C-17″), 12.0 (C-14′), 11.1 (C-10). All presented NMR data of **10** were in accordance with iristectorene B [59]. Therefore, the structure of **10** was determined as iristectorene B (Figure 1).

### 2.2. Effects of Compounds ***1***–***10*** on the Proliferation of Breast Cancer Cells

**Figure 2:**
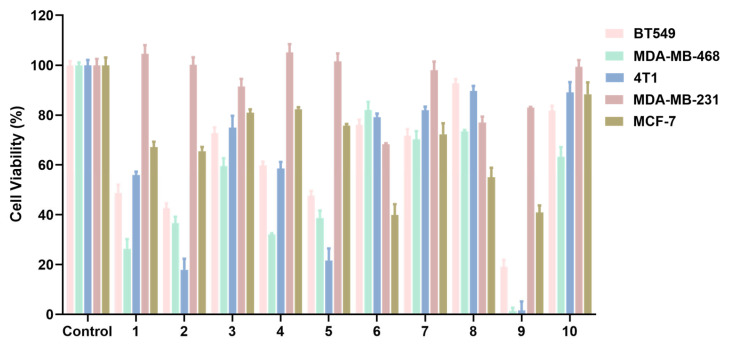
The anti-proliferation effects of compounds **1**–**10** on breast cancer cells treated with 50 μM.

The isolated compounds were evaluated for their antiproliferation effects on BT549, 4T1, MDA-MB-468, MDA-MB-231, and MCF7 cell lines treated with 50 μM by CCK-8 assay (Figure 2). The results show the different antiproliferation effects of compounds **1**–**10** on five kinds of breast cancer cells. Notably, compounds **9** and **10** were determined for the cytotoxicity of breast cancer cells for the first time. Among five kinds of breast cancer cells, compound **9** displayed the highest activity against the BT549, 4T1 MDA-MB-468, and MCF7 cell lines. In addition, the cytotoxicity of compound **10** clearly became weaker than compound **9** as a result of the substituent group of the long fatty chain. Consequently, compound **9** was chosen for subsequent mechanistic studies.

### 2.3. Compound ***9*** Inhibited TNBC Cell Proliferation

**Figure 3:**
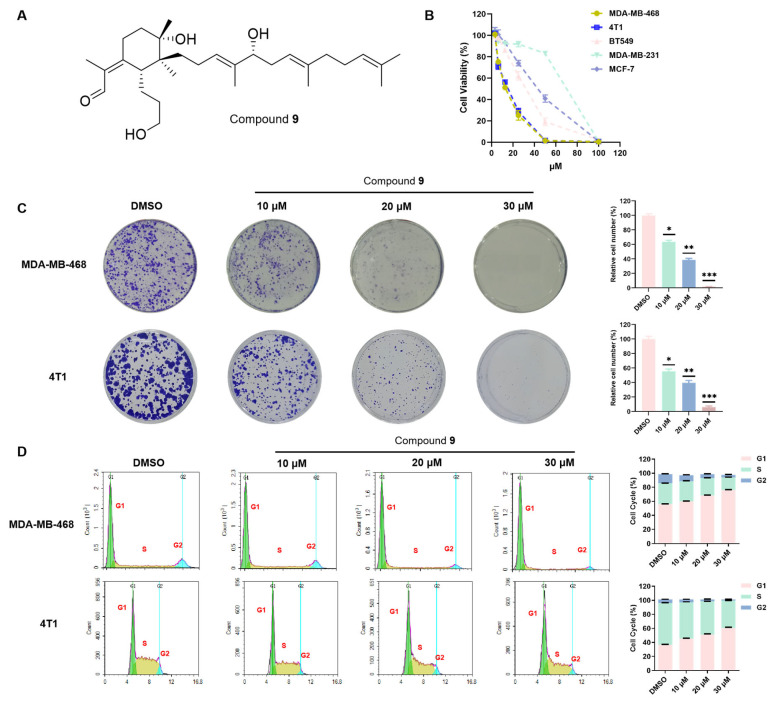
Compound **9** inhibited cell proliferation. (**A**) The structure of compound **9**; (**B**) CCK8 assays were performed to measure the antiproliferation potency of compound **9** against the BT549, 4T1, MDA-MB-468, MDA-MB-231, and MCF7 cell lines. (**C**) Colony formation assay of the MDA-MB-468 and 4T1 cells treated with 10, 20, and 30 μM of compound **9**. (**D**) Compound **9** influenced the cell cycle. The cell cycle was analyzed by PI staining and detected by flow cytometry. Representative images and quantifications of colonies are shown (* *p* < 0.05; ** *p* < 0.01; *** *p* < 0.001 vs. the control group).

Cell growth was inhibited in a dose-dependent manner by compound **9** in the BT549, 4T1, MDA-MB-468, MDA-MB-231, and MCF7 cell lines (Figure 3B), with IC_50_ values of 29.16 ± 1.54 μM, 13.51 ± 1.02 μM, 12.76 ± 0.82 μM, 59.80 ± 1.15 μM, and 39.83 ± 1.68 μM, respectively. Notably, the MDA-MB-468 and 4T1 cells were the most sensitive. Therefore, the MDA-MB-468 and 4T1 cells were chosen for subsequent experiments to further elucidate the preliminary antiproliferation mechanism of compound **9**.

To verify the above results, we conducted a colony formation assay to prove the effects of compound **9** on the proliferation of the MDA-MB-468 and 4T1 cells. The colony formation assay revealed treatment with compound **9** of 30 μM in the MDA-MB-468 and 4T1 cells compared with the control group could significantly reduce the overall number and size of colonies formed (*p* < 0.001) (Figure 3C). To further confirm the inhibitory effect of compound **9** on cell proliferation, we performed a cell cycle assay. Cells prepare for DNA replication in the G1 phase; the G2/M phase continuously allows preparation for mitosis. During the process of development, differentiation, or regression of growth factors, cells can enter the inactive phase G0 and then recover to G1 [60]. We detected the cell cycle by propidium iodide staining (PI staining). After the DNA inside the cells was stained with PI, the DNA content of the cells could be measured using flow cytometry. Then, based on the distribution of DNA content, cell cycle analysis can be performed. Compared with the control group, treatment with compound **9** of 20 or 30 μM in the MDA-MB-468 and 4T1 cells could prominently accumulate the cells in the G1 phase and decrease the S phase (Figure 3D). In summary, these results demonstrated that compound **9** inhibited the growth of MDA-MB-468 and 4T1 cells.

### 2.4. Compound ***9*** Suppressed Metastasis in the TNBC Cells

**Figure 4:**
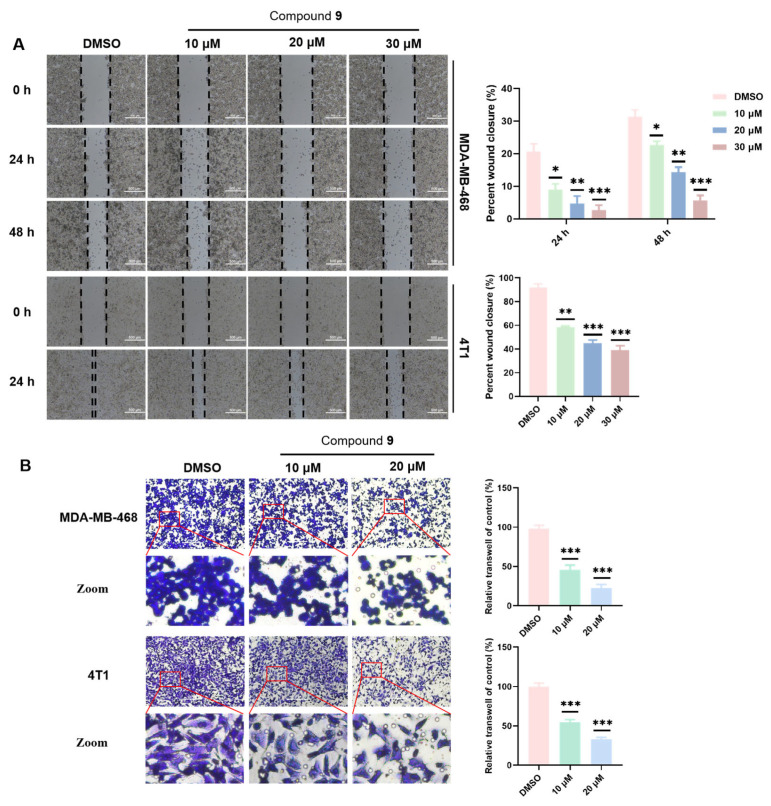
Compound **9** inhibited cell metastasis in the MDA-MB-468 and 4T1 cells. (**A**) The wound-healing assay detected the migration of MDA-MB-468 and 4T1 cells after different concentrations of compound **9** were used for treatment at different times. (**B**) The transwell assay detected the effect after different concentrations of compound **9** on the invasion ability of the MDA-MB-468 and 4T1 cells. Representative images and quantifications of the wound-healing assay and the transwell assay are shown. (* *p* < 0.05; ** *p* < 0.01; *** *p* < 0.001 vs. the control group).

Tumor metastasis is the main cause of poor prognosis in TNBC patients and poses a serious threat to them. Consequently, we conducted wound-healing assay and transwell assays to assess whether compound **9** was able to suppress MDA-MB-468 and 4T1 cell metastasis. As shown in the wound-healing assay, treatment with compound **9** of 30 μM in the MDA-MB-468 cells compared with the control group could significantly increase the percentage wound closure (%) (*p* < 0.001), but treatment with compound **9** of 20 μM in the 4T1 cells could markedly increase the percentage wound closure (%) (*p* < 0.001). In addition, the higher the concentration or the longer the treatment time of compound **9,** the higher the percentage of wound closure (%), which revealed that compound **9** could dose- and time-dependently inhibit migration in the MDA-MB-468 and 4T1 cells (Figure 4A). Similarly, the results of the transwell assay confirmed that compound **9** of 10 μM could significantly decrease the number of invaded MDA-MB-468 and 4T1 cells (*p* < 0.001) (Figure 4B), and compound **9** could dose-dependently inhibit invasion. In brief, these results demonstrated that compound **9** suppressed metastasis in the MDA-MB-468 and 4T1 cells.

### 2.5. Effect of Compound ***9*** on TNBC Cell Apoptosis

Apoptosis, programmed cell death, regulates the balance between cell growth and death. Tumor cells that evade apoptosis could promote metastasis [61]. Accordingly, we investigated cell apoptosis detection via Annexin V-FITC/PI staining and Hoechst staining. The Annexin V-FITC/PI staining detection kit makes use of FITC-labeled Annexin V as a probe to detect the occurrence of early cell apoptosis. PI cannot penetrate the intact cell membrane of normal or early apoptotic cells but can pass through the cell membrane of late apoptotic and necrotic cells. Therefore, PI is used to distinguish between surviving early cells and necrotic or late apoptotic cells. Hoechst 33342 staining is a solution suitable for staining the nucleus of living cells. The very bright blue fluorescence staining of the nucleus can be observed by a fluorescence microscope.

**Figure 5:**
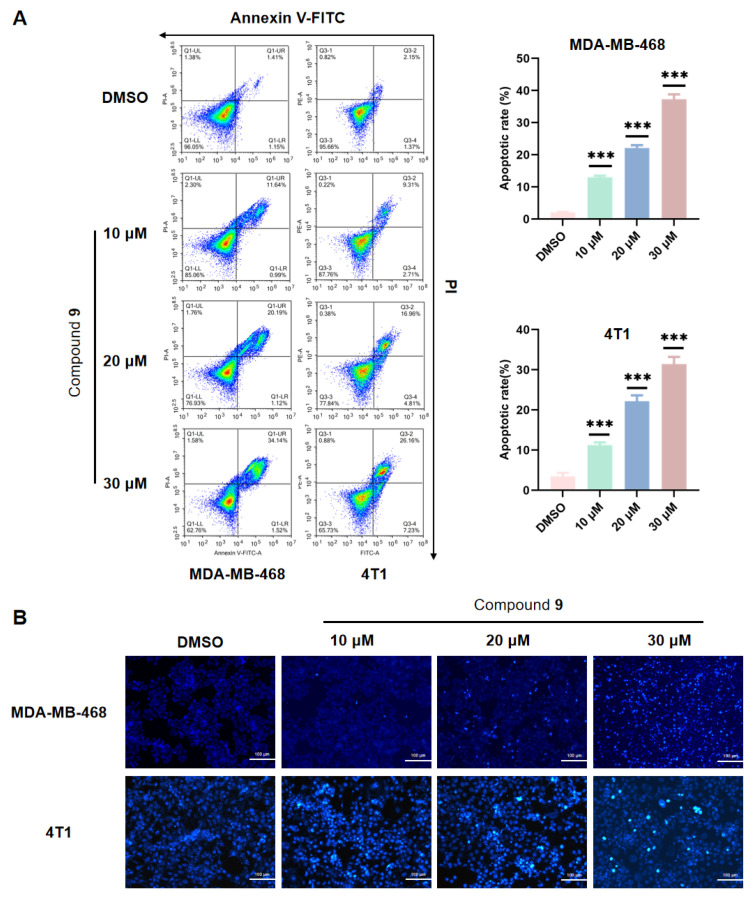
Compound **9** induced cell apoptosis in the MDA-MB-468 and 4T1 cells. The apoptotic cells were assayed by Annexin V-FITC/PI staining via flow cytometry. (*** *p* < 0.001 vs. the control group) (**A**) and Hoechst 33342 staining as observed by a fluorescence microscope (×200) (**B**).

In comparison with the control group, after treatment with compound **9** of 10 μM to 30 μM, the percentage of MDA-MB-468 and 4T1 cells in PI- and Annexin-V-FITC-positive cells was significantly increased via flow cytometry, which demonstrated that the apoptotic rate of the MDA-MB-468 and 4T1 cells was significantly increased (*p* < 0.001) (Figure 5A). The results indicated that compound **9** dose-dependently induced both early and late apoptosis in the MDA-MB-468 and 4T1 cells. Moreover, the results of Hoechst 33342 staining observed by a fluorescence microscope were consistent with Annexin V-FITC/PI staining. Compared with the control group, after treatment with compound **9** of 10 μM to 30 μM, the bright blue fluorescence became stronger and underwent typical morphological changes such as nucleus condensing and fragmentation (Figure 5B) in the MDA-MB-468 and 4T1 cells, which revealed that compound **9** could induce cell apoptosis. Overall, these results showed that compound **9** dose-dependently induced MDA-MB-468 and 4T1 cell apoptosis.

### 2.6. Effect of Compound ***9*** on Mitochondrial Membrane Potential Level

A decrease in mitochondrial membrane potential (MMP) levels is one of the characteristics of early apoptosis in tumor cells. To evaluate the role of mitochondria-induced cell death after treatment with compound **9**, we examined MMP by JC-1 staining in the MDA-MB-468 and 4T1 cells. Red fluorescence revealed JC-1 aggregation, which represented an intact MMP, while green fluorescence represented JC-1 monomers, indicating a dissipated MMP. The decrease in MMP could be easily detected by the change of JC-1 from red fluorescence to green fluorescence, and the change of JC-1 from red fluorescence to green fluorescence could also be used as a characteristic sign of cells’ early apoptosis.

**Figure 6:**
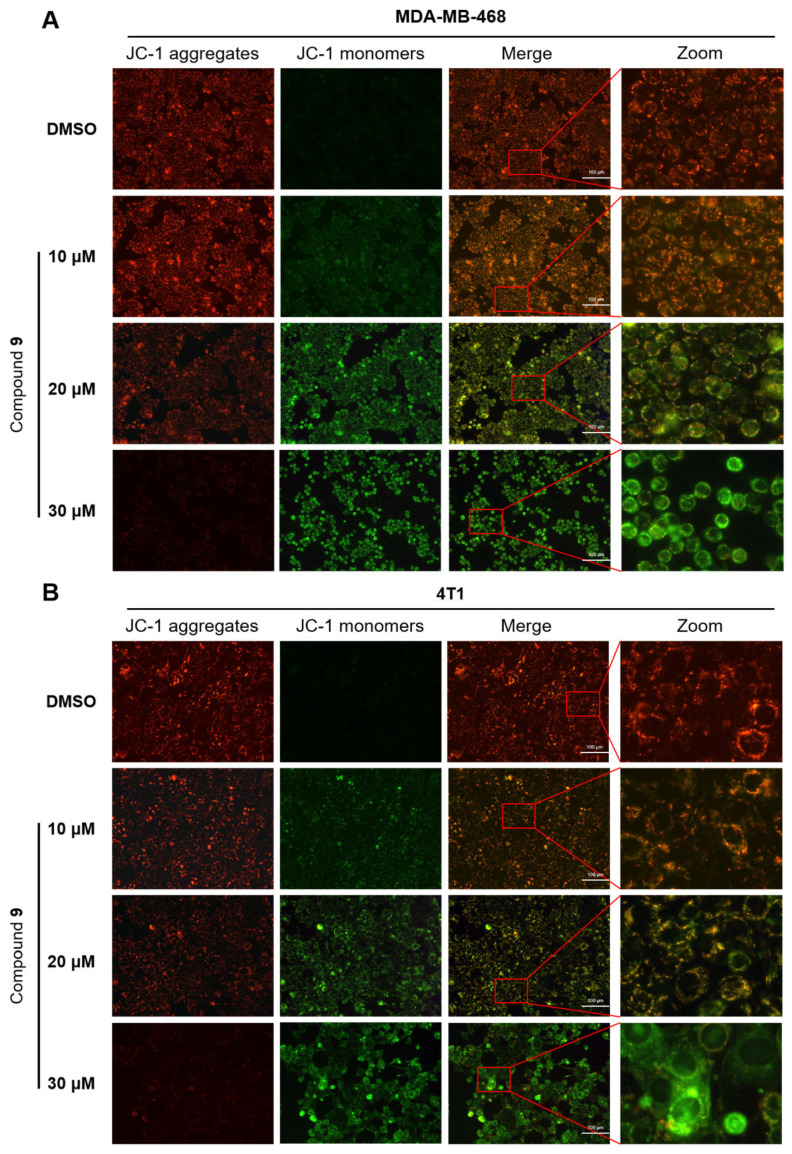
Compound **9** disrupted the MMP in the MDA-MB-468 and 4T1 cells. The MDA-MB-468 cells (**A**) and 4T1 cells (**B**) were treated with different concentrations of compound **9** and subjected to JC-1 staining. The MMP was detected by fluorescence (×200).

Compared to the control group, the JC-1 staining assay demonstrated that after treatment with compound **9** of 10 μM to 30 μM in the MDA-MB-468 and 4T1 cells, the red fluorescence became weaker, whereas, on the contrary, the green fluorescence became stronger (Figure 6), which revealed that the MMP levels decreased. As a consequence, these results demonstrated that compound **9** could dose-dependently disrupt MMP, which suggested that compound **9** induced early apoptosis in the MDA-MB-468 and 4T1 cells.

### 2.7. Effect of Compound ***9*** on ROS Level

Reactive oxygen species (ROS) are a heterogeneous group of highly reactive ions and molecules that are produced by metabolic reactions in mitochondria. ROS maintain homeostasis through the redox pathway, and increasing ROS levels are able to promote tumor cell death and limit cancer progression [62]. We investigated ROS level via an ROS assay kit, which is a reagent kit that makes use of a fluorescent probe of 2′,7′-dichlorodihydrofluorescein diacetate (DCFH-DA) for ROS detection. DCFH-DA itself does not have fluorescence and can freely pass through the cell membrane. After entering the cell, it can be hydrolyzed by esterases inside the cell to generate DCFH. However, DCFH cannot penetrate the cell membrane, making it easy for the probe to be loaded into the cell. Reactive oxygen species within cells can oxidize DCFH to produce fluorescent DCF. By detecting the fluorescence of DCF, the level of intracellular ROS can be determined.

**Figure 7:**
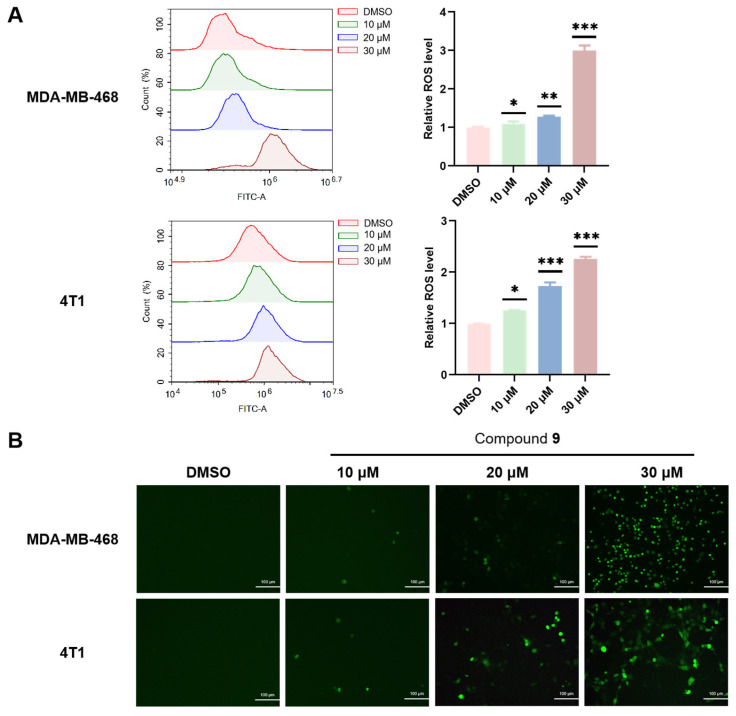
Compound **9** induced ROS production in the MDA-MB-468 and 4T1 cells. The ROS levels were measured by an ROS assay kit via flow cytometry and a fluorescence microscope. (**A**) Flow cytometry for analysis of intracellular ROS levels. (* *p* < 0.05; ** *p* < 0.01; *** *p* < 0.001 vs. the control group). (**B**) Representative images from the fluorescence microscope of intracellular ROS levels (×200).

DCF fluorescence demonstrated that at higher concentrations of treatment of compound **9** in the MDA-MB-468 and 4T1 cells through flow cytometry, the fluorescence became stronger which indicated that the ROS levels were higher (Figure 7A). In detail, compound **9** of 30 μM in the MDA-MB-468 cells and 20 μM in the 4T1 cells significantly increased the ROS levels (*p* < 0.001) compared to the control group. In addition, the results from the fluorescence microscope were identical; DCF fluorescence revealed a significant increase in ROS levels in a dose-dependent manner (Figure 7B). The above results indicated that compound **9** could facilitate ROS accumulation in the MDA-MB-468 and 4T1 cells and revealed that compound **9** induced oxidative stress and mitochondrial dysfunction during the apoptosis of the MDA-MB-468 and 4T1 cells.

In summary, ten compounds were isolated from the *rhizome* of *Belamcanda chinensis* (L.) DC, including four compounds (**1**, **5**, **7**, and **8**) first isolated from the genus *Belamcanda Adans*. nom. conserv. and six known compounds (**2**–**4**, **6**, **9**, and **10**). Ten compounds had antiproliferative effects against five breast cancer cell lines (BT549, 4T1, MCF7, MDA-MB-231, and MDA-MB-468). Among them, compound **9** had the best inhibitory effect against the 4T1 and MDA-MB-468 cells. In addition, antiproliferative effects, apoptosis induction, and potential molecular mechanisms of action were evaluated for the first time. It was demonstrated that compound **9** showed significant antiproliferative activity and inhibited metastasis in the MDA-MB-468 and 4T1 cells. Subsequently, Annexin V-FITC/PI showed an increase in apoptotic rates, and with Hoechst 33342 staining, significant changes in cell morphology were found after treatment with compound **9** of 10 μM to 30 μM in the MDA-MB-468 and 4T1 cells. As we know, depolarization of the mitochondrial membrane leads to the release of pro-apoptotic factors in mitochondria and the activation of apoptotic proteases and ultimately induces apoptosis. Excessive production of ROS leads to oxidative damage to important cellular molecules and structures, resulting in mitochondrial dysfunction and activation of endogenous apoptotic pathways and ultimately causing cell apoptosis [63]. As mentioned earlier, the changes in the mitochondrial membrane potential level and the ROS level revealed that compound **9** could induce apoptosis in MDA-MB-468 and 4T1 cells for the first time. In conclusion, all of the results revealed compound **9** may be a leading compound for triple-negative breast cancer to merit further study.

## 3. Materials and Methods

### 3.1. Separation and Purification

*Belamcandae chinensis rhizoma* was purchased from Heshun Tang Pharmacy in Shenzhen, China, in October 2020 and identified by Dr. Li Chenyang from Shenzhen University. Approximately 19 kg *Belamcandae chinensis rhizoma* was ground into a fine powder and extracted with 95% ethanol by refluxing extraction three times, each time for 2 h. The extraction solution was combined, the pressure was reduced, and it was evaporated to a certain volume, and then the crude extract was further fractionated through macroporous resin column chromatography and eluted successively with 45%, 75%, and 95% ethanol, which was brought to dryness under reduced pressure, yielding dried 45% (3.461 kg), 75% (350 g), and 95% (341 g) ethanol extract, respectively. The cytotoxicity assay of the three extracts (Figure S1) indicated that the 75% ethanol extract showed the highest activity. Therefore, isolation for the active compounds from the 75% ethanol extract of *Belamcandae chinensis rhizoma* was carried out.

The 75% ethanol extract was fractionated by silica gel column chromatography using petroleum ether: ethyl acetate (50:1, 20:1, 10:1, 4:1, 2:1, and 1:1) and methanol successively, and it produced seven sub-fractions (Fr. A−Fr. G). Compound **8** (30.0 mg) was purified by crystallization from Fr. B with methanol. Compound **10** (81.3 mg) was purified by semi-preparative RP-HPLC (YMC-Pack ODS-A 250 mm × 10 mm; flow rate: 3 mL/min) under the following gradient: MeOH−H_2_O (95:5 to 100:0 over 30 min) from Fr. C. Fr. D was separated by medium- and low-pressure ODS column chromatography, and gradient elution was performed with MeOH−H_2_O (50%−100%). The obtained compounds, **1** (194.0 mg), **2** (376.0 mg), **4**, (39.7 mg), **7** (32.4 mg), and **3** (20.0 mg), were purified by crystallization with methanol. In addition, compound **5** (39.0 mg) was isolated with 45% methanol aqueous solution using semi-preparative RP-HPLC (YMC-Pack ODS-A 250 mm × 10 mm; flow rate: 3 mL/min) from Fr. D. Subsequently, compound **9** (179.0 mg) was separated with 71% methanol aqueous solution by semi-preparative RP-HPLC (Phenomenex Luna C18 (2) 250 mm × 10 mm, flow rate: 3 mL/min) from Fr. E. Finally, compound **6** (4.0 mg) was purified by Sephadex LH−20 (CH_2_Cl_2_−MeOH, 1:1) from Fr. F. Compounds **1**–**10** were confirmed by spectroscopic data, and they were rhapontigenin, trans-resveratrol, 5,7,4′-trihydroxy-6,3′,5′-trimethoxy-isoflavone, irisflorentin, 6-hydroxybiochannin A, iridin S, pinoresinol, 31-norsysloartanol, isoiridogermanal, and iristectorene B, respectively.

### 3.2. Cell Culture

Five breast cancer cell lines: MDA-MB-468, MDA-MB-231, BT549, MCF7, and 4T1 were obtained from the American Type Culture Collection (ATCC, Rockville, MD, USA). The MDA-MB-468 cells were routinely cultured in RPMI-1640 (Corning, New York, NY, USA), and the 4T1 cells were cultured in high-glucose Dulbecco’s modified Eagle’s medium (DMEM) (Corning), which was supplemented with 10% fetal bovine serum (FBS) (Trans Gen Tech, Beijing, China) and penicillin/streptomycin (100 unit/mL, Gibco, Waltham, MA, USA) in 5% CO_2_ at 37 °C.

### 3.3. Cell Viability Assay

The cells were seeded in 96-well plates with 3 × 10^4^ cells and 1 × 10^4^ cells per well, incubated in a 37 °C incubator with 5% CO_2_ added overnight. The cells were treated with compounds at different concentrations for 48 h in 5% CO_2_ at 37 °C. The original medium was discarded, and each well was filled with 100 μL of culture medium containing 10% CCK-8 and incubated for 1 h at 37 °C in an incubator, and then the absorbance at 450 nm was detected with a microplate reader (POLARstar Omega, Washington, DC, USA).

### 3.4. Colony Formation

The cells were seeded in a 6-well plate (2 × 10^3^ cells/well) and cultured overnight in 5% CO_2_ at 37 °C for 7 days. When the colonies formed, they were treated with compounds at different concentrations, and then they were cultured in a 37 °C incubator with 5% CO_2_ for 10 days. The fresh complete culture medium was replaced every other day. When the colonies were cultured to the naked eye, the colonies were fixed with 4% paraformaldehyde and stained with 0.1% crystal violet solution.

### 3.5. Cell Cycle Analysis

The cells were seeded in a 6-well plate and cultured overnight in 5% CO_2_ at 37 °C. Then, the cells were treated with drugs of different concentrations for 48 h in 5% CO_2_ at 37 °C. The original medium was discarded, and the cells were harvested and fixed by 70% ice-cold (*v*/*v*) ethanol overnight at 4 °C. After that, the fixed cells were centrifuged and washed with cold phosphate-buffered saline (PBS), and then, they were further resuspended in a solution of 0.5 mL propidium iodide (PI) and RNAase staining buffer for 30 min at 37 °C in the dark. The cells were determined by an Agilent NovoCyte Flow Cytometer, and the data were analyzed by NovoExpress software (version 1.6.1).

### 3.6. Wound-Healing Assay

The cells were seeded in a 6-well plate in a 37 °C incubator with 5% CO_2_, and after the cells reached confluency, a sterile pipette tip was used to draw parallel lines in each cell, and the cells were washed with PBS. In succession, a medium with 2% FBS was added with compounds at different concentrations, and a microscope was used to take pictures at 0 h, 24 h, and 48 h to analyze the results.

### 3.7. Transwell Assay

The cells were resuspended in 200 μL FBS-free medium into the upper chamber, and 600 μL complete medium with compounds at different concentrations was added into the lower chamber. After incubating for 48 h, the cells were fixed with 4% paraformaldehyde and stained with 0.1% crystal violet solution. The cells were photographed and counted under the microscope.

### 3.8. Annexin V-FITC/PI Double Staining Assay

Apoptosis was detected by an Annexin V-FITC/PI staining kit. The cells were seeded in a 6-well plate and cultured overnight in 5% CO_2_ at 37 °C. Then, the cells were treated with drugs of different concentrations for 48 h in 5% CO_2_ at 37 °C. The original medium was discarded, and the cells were collected and washed twice with cold PBS. The collected cells were dispersed with 100 μL 1 × binding buffer, and then, they were incubated with 5 μL Annexin V-FITC and 10 μL PI staining solution for 15 min at room temperature in the dark. The stained cells were detected by an Agilent NovoCyte Flow Cytometer, and the data were analyzed by NovoExpress software (version 1.6.1).

### 3.9. Hoechst 33342 Staining

The cells were seeded in a 6-well plate and cultured overnight in 5% CO_2_ at 37 °C. After the cells were treated with compounds at different concentrations for 48 h in 5% CO_2_ at 37 °C, the cells were washed twice with PBS and then stained with Hoechst 33342 fluorescent dye for 30 min at 37 °C in the dark. Then, using a fluorescence microscope, morphological changes in the nucleus were examined.

### 3.10. JC-1 Staining

Mitochondrial membrane potential (MMP) was determined by an MMP assay kit. Briefly, the cells were seeded in a 6-well plate and cultured overnight in 5% CO_2_ at 37 °C. After the cells were treated with compounds of different concentrations, the medium was removed and washed with sterile PBS, and then, the cells were stained with a JC-1 fluorescent probe for 30 min at 37 °C in the dark. Thereafter, the cells were washed with 1 × JC-1 buffer solution three times, and the cells were pictured via fluorescence microscopy.

### 3.11. Measurement of Intracellular ROS Levels

The intracellular ROS levels were detected with an ROS assay kit. The cells were seeded in a 6-well plate and cultured overnight in 5% CO_2_ at 37 °C. After the cells were treated with compounds at different concentrations, the medium was removed and washed with sterile PBS, and, on one hand, incubated with a DCFH-DA probe for 20 min at 37 °C in the dark, and then, the cells were washed with PBS three times, and the cells were pictured via fluorescence microscopy. On the other hand, the cells were collected, stained with a DCFH-DA probe, and ROS were determined by an Agilent NovoCyte Flow Cytometer, and the data were analyzed by NovoExpress software (version 1.6.1).

### 3.12. Statistical Analysis

All cell culture assays were performed in triplicate. The data were analyzed and processed by GraphPad Prism 9.0 software. The data were expressed in the form of the mean ± SD. Differences between the groups were compared with the statistical significance by a *t*-test. *p* < 0.05 represents a statistical difference.

## 4. Conclusions

In this study, four compounds (**1**, **5**, **7**, and **8**) were first isolated from the genus *Belamcanda Adans*. nom. conserv., and six known compounds (**2–4**, **6**, **9**, and **10**) were isolated from the rhizome of *Belamcanda chinensis* (L.) DC. The antiproliferative effects against five tumor cell lines (BT549, 4T1, MCF7, MDA-MB-231, and MDA-MB-468) suggested that compound **9** had the best inhibitory effect against the MDA-MB-468 and 4T1 cells. Subsequently, we found compound **9** could inhibit the proliferation and metastasis of MDA-MB-468 and 4T1 cells and induce cell cycle arrest in the G1 phase. Moreover, we found that compound **9** could downregulate mitochondrial membrane potential and produce excessive ROS to induce apoptosis of the MDA-MB-468 and 4T1 cells for the first time. In conclusion, our findings demonstrate that compound **9** may be a leading compound for triple-negative breast cancer to merit further study.

## Data Availability

Not applicable.

## Supplementary Materials

The following supporting information can be downloaded at: https://www.mdpi.com/article/10.3390/molecules28124715/s1, Figure S1. The antiproliferative effects against seven tumor cells (4T1, MDA-MB-231, BT549, MDA-MB-468, HepG2, Hela, and A549) for 100 μg/mL total extract of *Belamcandae chinensis rhizoma* and its subfraction.

## Sample Availability

Samples of the compounds are available from the authors.
