# Supplementary material for: Cytotoxic Compounds from Belamcanda chinensis (L.) DC Induced Apoptosis in Triple-Negative Breast Cancer Cells
Source: Molecules. 2023 Jun 12;28(12):4715. doi: 10.3390/molecules28124715 (PMC10305019; doi:10.3390/molecules28124715)
Supplement: Supplementary file 1 [file molecules-28-04715-s001.zip › molecules-2425493-supplementary.pdf]

Supplementary Information

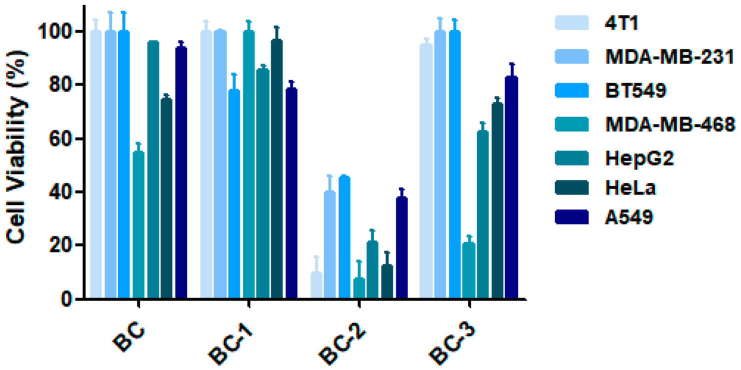

Figure S1. The antiproliferative effects against seven tumor cells (4T1, MDA-MB-231, BT549, MDA-MB-468, HepG2, Hela and A549) for 100 μg/mL total extract of *Belamcandae chinensis rhizoma* and its subfraction. BC: Total extract of *Belamcandae chinensis rhizoma*; The fraction from macroporous resin column chromatography eluted with 45% ethanol extract (BC-1), 75% ethanol extract (BC-2), 95% ethanol extract (BC-3) of total extract of *Belamcandae chinensis rhizoma.*
